# Door-to-Balloon Time and Mortality in STEMI With Cardiogenic Shock: A Nationwide Registry

**DOI:** 10.1016/j.jacasi.2024.03.002

**Published:** 2024-04-23

**Authors:** Yuichi Saito, Taku Inohara, Shun Kohsaka, Takashi Muramatsu, Hideki Ishii, Kyohei Yamaji, Tetsuya Amano, Yoshio Kobayashi, Ken Kozuma, The J-PCI Registry Investigators

**Affiliations:** a Chiba University Graduate School of Medicine, Chiba, Japan; b Keio University School of Medicine, Tokyo, Japan; c Fujita Health University Hospital, Toyoake, Japan; d Gunma University Graduate School of Medicine, Gunma, Japan; e Kyoto University, Kyoto, Japan; f Aichi Medical University, Nagakute, Japan; g Teikyo University Hospital, Tokyo, Japan

Door-to-balloon time serves as a readily available and pragmatic quality metric for managing ST-segment elevation myocardial infarction (STEMI).^1^ Although door-to-balloon time intuitively plays a role when complicated by cardiogenic shock (CS), previous studies have reported mixed results concerning the association between delays in primary revascularization procedures and mortality in patients with STEMI with CS.^2^^,^^3^

The Japanese PCI (J-PCI) registry covers approximately 90% of all percutaneous coronary intervention (PCI) procedures performed in Japan.^4^ The study protocol for the J-PCI registry was approved by a third-party central ethics committee (Clinical Research Promotion Network Japan, Osaka, Japan). Between January 2019 and December 2021, 734,379 PCI procedures at 1,190 hospitals were registered in the J-PCI. We enrolled patients with STEMI who were undergoing primary PCI. Patients aged <20 or >100 years and missing data on in-hospital outcomes, CS, cardiac arrest (CA), and door-to-balloon time were excluded. We did not include patients with a door-to-balloon time <15 or >180 minutes.^1^ Patients with CA but without CS were also excluded. The primary endpoint was in-hospital mortality.

Of 100,672 patients included, 13,222 (13.1%) had cases complicated by CS, and 5,278 (40.0%) also had CA. Patients with CS were more likely to be older and women and to have chronic kidney disease, peripheral artery disease, and previous PCI and coronary artery bypass grafting. Mechanical circulatory support (MCS) devices were used in 7,000 (52.9%) patients with CS. Overall, the median door-to-balloon time was 70 minutes (Q1-Q3: 54-89 minutes), and 9,070 of 13,222 (68.6%) patients had a door-to-balloon time ≤90 minutes. The door-to-balloon time was longer in patients with CS than in those without CS (77 minutes [Q1-Q3: 58-100 minutes] vs 69 minutes [Q1-Q3: 53-87 minutes]; *P* < 0.001), and the presence of CS and CA was associated with increased door-to-balloon time.

**Figure 1:**
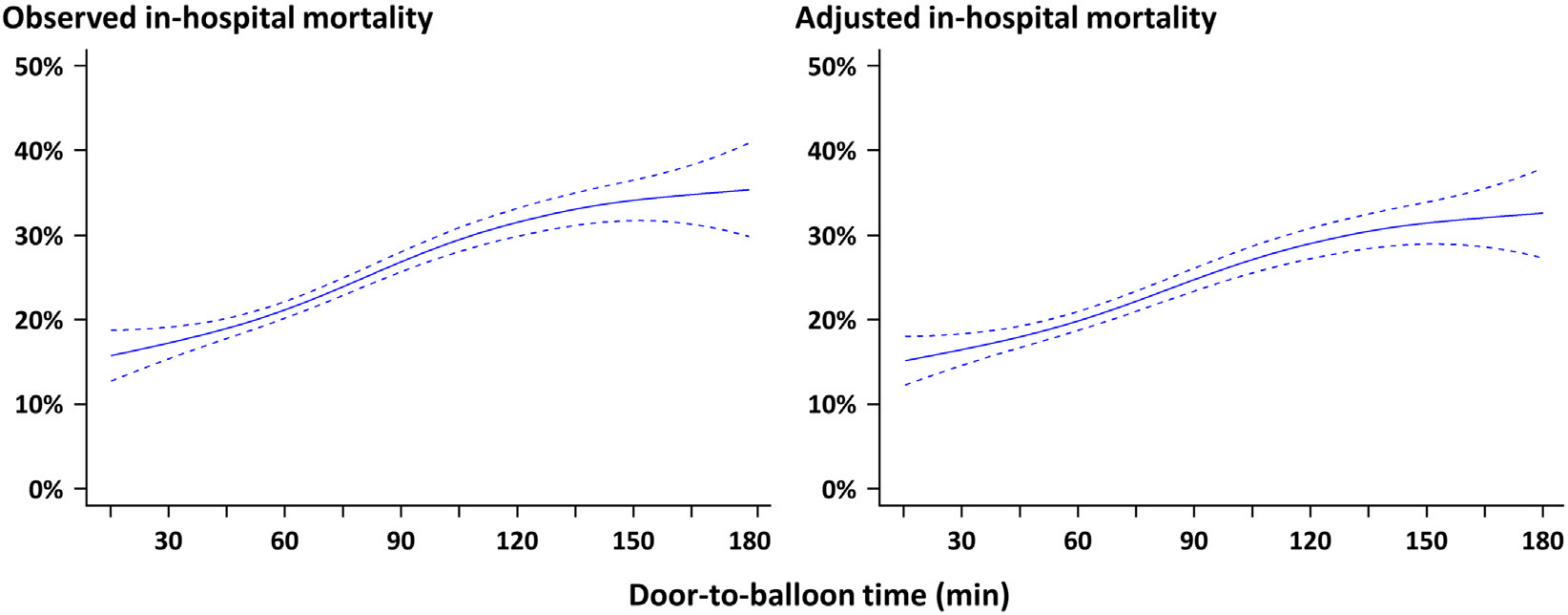
Relationship of Door-to-Balloon Time With Observed and Adjusted In-Hospital Mortality Longer door-to-balloon time was associated with increased observed and adjusted in-hospital mortality. The probability of in-hospital mortality was adjusted with age, sex, chronic kidney disease, hemodialysis, and chronic obstructive pulmonary disease by using logistic regression analysis. Dashed lines indicate 95% CIs. For the adjustment, we incorporated median values for continuous variables (age 71 years) and mode values for categorical variables (male sex, no chronic kidney disease, no hemodialysis, and no chronic obstructive pulmonary disease) as a method of standardization.

In-hospital mortality was higher in patients with CS and CA, followed by those with CS and no CA and those without CS (36.7% vs 17.0% vs 2.3%; *P* < 0.001). Notably, door-to-balloon time had a positive correlation with observed and adjusted in-hospital mortality in patients with STEMI and CS (Figure 1). Multivariable analysis identified shorter door-to-balloon time as a factor associated with better survival (OR: 1.07; 95% CI: 1.05-1.09; *P* < 0.001). The door-to-balloon time was significantly associated with in-hospital mortality in the subgroups of CA, MCS device use, and hospital volume, and the effect of shorter door-to-balloon time on survival was more pronounced in patients without CA or MCS device use, although the differences in relative mortality between the presence and absence of CA or MCS device use were small.

Our data demonstrated that a longer door-to-balloon time was associated with higher in-hospital mortality, even after multivariable adjustment. In the multicenter, prospective FITT-STEMI (Feedback Intervention and Treatment Times in ST-Elevation Myocardial Infarction) study, 1,530 of 12,306 (12.4%) patients with STEMI had CS with or without CA.^3^ FITT-STEMI showed that first medical contact-to-balloon time was delayed by 6.9 minutes in patients with STEMI complicated by CS, and shorter ischemic time was associated with better in-hospital survival, findings in line with our results.^3^ The present study confirmed the prognostic impact of ischemic duration by using door-to-balloon time in patients with STEMI and CS and may provide a benchmark for future studies. Given the observational study design, our results do not indicate a causal relationship between door-to-balloon time and in-hospital mortality. Additionally, our results should be interpreted with a notion of survivor bias.^5^ Whether actions to shorten door-to-balloon time reduce mortality in patients with STEMI complicated by CS deserves further investigation.
